# Management of muscular dystrophy during osteoarthritis disorder: A topical phytotherapeutic treatment protocol

**DOI:** 10.22088/cjim.10.2.183

**Published:** 2019

**Authors:** Apurba Ganguly

**Affiliations:** 1 Department of Research and Development, OPTM Research Institute, India

**Keywords:** Muscular dystrophy, Specific biomarkers, Phytotherapy, Normalized serum markers, OAD with muscular dystrophy.

## Abstract

**Background:**

Creatine kinase-muscle (CK-MM) and aldolase A (AldoA) levels are proven to be realistic biochemical markers to detect muscular dystrophy during osteoarthritic disorders (MD-OADs). The aim of this study is to normalize the MD-OADs characterized by muscle weakness, atrophy, inflammatory disorders, pain with chronic arthropathy by specialized topical phytotherapeutic treatment.

**Methods:**

Baseline data were collected and evaluated from 153 patients, aged 59.89±11.37years, and suffering with MD-OADs for 7.89±1.90 years. Serum CK-MM and aldoA levels were measured at baseline and after a six- week treatment using the appropriate kits. All patients underwent standardized physical, radiographic examinations and completed a questionnaire. All the patients were treated with topical application of phytoconstituents from the extracts of seven Indian medicinal plants namely *Cissus quadrangularis*, *Calotropis gigantea*, *Zingiber officinalis*, *Rosemarinus officinale*, *Boswellia serratia, Curcuma longa and* *Withata somnifera *mixed with sesame oil and beehives wax for six-week.

**Results:**

The elevated levels of biomarkers, CK-MM and aldoA, were reduced to their mean±SEM values 82.77±1.32 and 4.94±1.30U/L, respectively at the end of six-week treatment and the improvements of deranged anatomical features, Pearson’s correlation coefficients, international-approved pain related abnormalities (VAS, WOMAC, KPS and KOOS) and reduction of weight at the end of treatment were all highly significant (p<0.0001).

**Conclusion:**

It is firmly confirmed that MD-OADs resulted with the elevated levels of CK-MM, and AldoA, along with deranged anatomical features (KGB, DTM, DCM, DAP, DBP, SLR, KFS and KES) and pain related parameters (VAS, WOMAC-index, KPS, KOOS and BMI) can be successfully normalized by topical phytotherapeutic treatment protocol.

According to Newman (1), muscular dystrophy (MD) is a group of disorders in which progressive loss of muscle mass due to interference with the production of muscle protein called dystrophin, (first identified in 1987 by Louis M. Kunket), essential for building and repairing muscles, lead to muscular wasting, muscle weakness and degeneration, whereas osteoarthritic disorders (OADs) are commonly characterized by the potential loss of joint cartilage as well as osteophyte formation, connective tissue damage, pain symptoms, joint effusion, restricted movement of joints, tenderness and crepitus in the joints (2-6). But several muscle degenerations are leading to muscular dystrophy also matter of great concern during the progression of OADs.

Researches have been well-documented with regard to the impairment of different muscles during MD-OADs (7-10) and also it was reported that women are more vulnerable than men (7, 9, 11).

There are several types of MD such as Duchenne muscular dystrophy (DMD) and Becker muscular dystrophy (BMD) which are very common. But none of DMD or BMD is involved in MD-OAD as it does not occur relating to their phenomenon (12). The normal symptoms of MD-OAD include: a waddling gait; pain and stiffness in the muscles; difficulty with running and jumping; difficulty in sitting and standing; frequent falls; shortening of muscles and tendons which lead to further limiting movement of joints, cartilage damage effusion, bone hypertrophy and lordosis (13). Both DMD and BMD are associated with a heart condition called cardiomyopathy which includes an irregular heartbeat, shortness of breath, fatigueless etc. developed in childhood or in adolescence but the above phenomena are not indicated in MD-OAD. Interestingly, it is to be noted that these two characteristics during DMD and BMD differed in their severity, age of onset, and rate of progression, as in DMD, muscle weakness tends to appear in early childhood while BMD occurs later in childhood or in adolescence with slower rate of progression, but MD-OAD occurs >50 years of age with muscle atrophy and weakness along with chronic arthropathy. Moreover, OADs are closely related to sarcopenia in which reduction of muscle mass was observed followed by failure of muscle strength and functional ability (14-16). Generally, estimation of serum aldolase level helps to diagnose MDs, neuromuscular disorders, and wasting of skeletal muscle (17-20). From past study by Sibley and Lehninger (21), it was reported that hyperaldolasaemia leads to MD. Previously, MD was identified by knowing elevated level of aldolase in serum, but researchers have found that the estimation of both muscle creatine kinase as well as aldolase levels in combinations can be suitable biochemical markers to detect MD along with skeletal muscle damage (22). From decade, the estimation of creatine kinase-muscle (CK-MM), which is found to be elevated and can be estimated through CPK-MM isoforms (23-28). In this context, several researchers have suggested muscular damage along with osteoarthritis that can be treated through sports and exercise for the therapy of muscular weakness, functional disability, etc. (29-31). Till date no medication was reported to prevent or reverse MD-OADs except pain relief and prevent inflammation by non-steroidal anti-inflammatory drugs (NSAIDs) or steroid drugs (32). But their use is associated with increased gastrointestinal and cardiovascular risk (33). But no specific treatment is available for MD-OADs which can reduce the biochemical parameters such as CK-MM and aldoA representing muscle degeneration, bone erosion and skeletal muscles damage. 

Moreover, the author has already developed a unique deranged anatomical measuring protocol to identify quantum of damage of muscles and tissues and also other biomarkers such as calcium, phosphorus, ratio of calcium and phosphorus and level of parathyroid hormone to identify the condition of damage bone during MD in OAD patients (28, 34-35). Recently the author has established the diagnostic protocol for MD-OADs evidenced by biochemical and anatomical parameters (36). It has been attempted earlier that increased CK-MM and AldoA levels in blood decreased after completion of topical application of natural compounds (phytochemicals) extracted from seven medicinal plants such as Calotropis gygantea (root and leaves), Cisssus quadrangularis (whole plant), Zingiber officinale (rhizome), Rosmarinus officinalis (leaves and flowers), Boswellia serrata (resin), Curcuma longa (rhizome) and Withania somnifera (root) (37-39). According to Ganguly A (37-38), this is a novel technique and for the first time it was observed with the help of aqueous phytoextraction as remedial measures. 

The present study was attempted for normalization of MD-OADs by analyzing the decreasing serum levels of CK-MM and aldoA in the patients before and after the treatment using phytoconstiuents extracted from the abovementioned seven plants mixed with sesame oil and beehive wax by specialized treatment protocol which have not been studied yet. 

## Methods

**Recruitment of patients: **From nine center of OPTM Health Care (P) Ltd 315 Indian patients (222 females and 93 males), aged 40 to 70 years old were selected for the study from July 2017 to December 2017, based on the sign and symptoms as described in earlier studies (27). The total number of 153 patients suffering for more than five years with MD-OADs was selected using exclusion criteria summarized in (table 1). The study protocol was evaluated and approved by the OPTM Research Institute Ethics Committee. An Institutional Review Board-approved consent form for the physical examinations, blood sample collections and other radiological examinations required for the study was signed by all the patients in the first phase of the screening procedure.

**Table 1:** Exclusion criteria during selection of patients

**Out of 315 patients, 162 patients (114 females and 48 males) were excluded.**
**After check-up**
112 patients (86 females and 26 males) were excluded for the following reasons with: cuts, wounds or any type of chronic skin disease (12 females and 4 males). parallel multiple drug dependence such as NSAIDS, corticosteroids etc. (14 females and 4 males). surgical implants (8 females and 3 males). a pacemaker (9 females and 2 males). a history of cancer, including caranomatosis and granulocytic leukemia (11females and 3 males). a history of severe neurological diseases (12 females and 2 males) a history of chronic liver, kidney and heart diseases ( 12 females and 4 males) and patients who did not agree to a physical evaluation (8 females and 4 males)
B. 34 patients (17 females and 17 males) were also additionally excluded for having another pathological condition that could explain the existing symptoms such as: rheumatic diseases, osteochondritis diseases, inter-articular fractures, congenital dysplasia, radicular syndrome, joint symptoms caused by malignant tumors, Baker’s cyst, Perthes disease, Plica syndrome, dermatomyositis and polymyositis diseases, iliopectineal or trochanteric bursitis, ischemic bone necrosis ligament or meniscus damage.
**Discontinued (dropped out) during treatment**
16 patients (11 females and 5 males) discontinued their treatment in the clinic because of : problems of transportation; shortage of helping hands at old age; unable to apply medicines at home, twice/thrice a day and transferring the jobs in other city as under: 7 patients after a week (3 females and a male) 7 patients after the second week (4 females and 2 males) 6 patients after the third week (2 females and a male) 5 patients after the mid of fourth week (2 females and a male)

**Study design: **After the analysis of exclusion criteria, each patient of 153 completed a questionnaire, providing details regarding demographics, medical history, nutritional status, ethnic barriers and work status at the baseline and summarized in table 2. 

**Evaluation of anatomical features: **Physical examinations were evaluated at the baseline and at the end of 6 weeks of post treatment including anatomical measurements as per established protocol of Ganguly (27) and range of motions (ROMs) was used in accordance with the American Academy of Orthopedic Surgeons (AAOS) (40).

**Table 2:** Demographic data and baseline characteristics of the study subjects

**Characteristic**	**Combined**	**Female**	**Male**
No of subjects (%)	153	108 (70.59)	45 (29.41)
Mean age (SD) in years	59.89 ( 11.37)	59.38 (10.21)	61.12 (13.68)
Mean weight (SD) in kg	69.71 (5.05)	69.12 (5.11)	71.13 (6.12)
Mean height (SD) in meter	1.58 (0.92)	1.55 (0.88)	1.67 (0.93)
Mean BMI (SD) in kg/m²	27.86 (6.94)	28.80 (6.64)	25.59 (7.11)
Mean symptom duration in years (SD)	7.89 (1.90)	7.78(1.78)	8.12 (2.11)
**Indian ethnic group (%)**			
Bengali	65(42.48 )	54 (50.00 )	11 (24.44 )
Gujrati	12 (7.84 )	7 (6.48 )	5 (11.11 )
Marwaree	14 (9.15 )	7 (6.48 )	7(15.55 )
Marathi	13 (8.51 )	8 (7.41 )	5 (11.11 )
Tamil	15 (9.80 )	11 (10.18 )	4 (8.89 )
Punjabi	13( 8.51)	7(6.48 )	6 (13.33 )
Shindhi	11 (7.18 )	8 (7.41 )	3 (6.68 )
North East India	10 (6.53 )	6 (5.56 )	4 (8.89 )
**Dietary habits (%) **			
Vegetarian	97 (63.40 )	71 (65.74 )	26 (57.78 )
Non- vegetarian	56 (36.60 )	37 (34.26 )	19 (42.22 )
**Other habits**			
Smoking	45 (29.41)	22 (20.37 )	23 (51.11 )
Drinking alcohol	14(9.15)	6 ( 5.55)	8 (17.78 )
Drinking tea and coffee	69 (45.10 )	57 (52.78 )	12 (26.67 )
Chewing tobacco	25 (16.34 )	23 (21.30 )	2 (4.44 )
**Analysis of radiological reports (%)**			
KOA in right knee with osteophytes	51 (33.33 )	35 ( 32.41 )	16( 35.56 )
KOA in left knee with osteophytes	57 (37.25 )	39 (36.11 )	18 (40.00 )
Degenerative changes in lumber region	45 (29.42 )	34 (31.48 )	11 (24.44 )
**Work status (%)**			
Employed fulltime	34 (22.22 )	12 (11.11 )	22 (48.89 )
Employed part time	7 (4.57 )	4 (3.70 )	3 (6.67 )
Housewife / Home- maker	66 (43.15 )	66 (61.11 )	-
Retired	18 (11.76 )	10 (9.26 )	8 (17.78 )
Self employed	28 (18.30 )	16 (14.82 )	12 (26.66 )
**Marital status (%)**			
Single	8 ( 5.23)	3 ( 2.78)	5 (11.11 )
Married	111 (72.55 )	86 (79.63 )	25 (55.55 )
Separated	8 (5.23 )	3 (2.78 )	5 (11.11)
Divorced	11 (7.19 )	5 (4.63 )	6 (13.34 )
Widowed	15 (9.80 )	11 (10.18 )	4 (8.89 )
**Multiple complaints or comorbiditie**s** (%)**			
Constipation	147 (96.08 )	105 (97.22 )	42 (93.33 )
Acidity and reflux	148 (96.73 )	104 (96.30 )	44 (97.78 )
Insomnia	112 (73.20 )	80 (74.07 )	32 (71.11 )
Varicose veins	81 (52.94 )	59 (54.63 )	22 (48.89 )
Urinary incontinence	89 (58.17 )	60 (55.55 )	29 (64.44 )
Crepitus during knee flexion	111 (72.55 )	96 (88.89 )	15 (33.33 )
Morning stiffness (<30 min.)	119 (77.78 )	102 (94.44 )	17 (37.78 )
**Measures taken to diminish pain and inflammation (%)**			
Knee cap uses	88 (57.52 )	51 (47.22 )	37 (82.22 )
Lumbar belt uses	25(16.34 )	15 (13.89 )	10 (22.22 )
Paracetamol and NSAID use	142 (92.81 )	99 (91.67 )	43 (95.56 )
Arthrocentesis (four months ago)	11 (7.19 )	3 (2.78 )	8 (17.78 )
Use of hyaluronic acid injection	25 (16.34 )	14 (12.96 )	11 (24.44 )
Use of corticosteroid injection	37 (24.18 )	25 (23.15 )	12 (26.67 )
Massage with herbal or other gels	144 (94.12 )	103 (95.37 )	41 (91.11 )
Homeopathic treatment	149 (97.38 )	107 (99.07 )	42 (93.33 )
Ayurvedic treatment	148 (96.73 )	104 (96.30 )	44 (97.78 )
Stick/walker use	25 (16.34 )	15 (13.89 )	10 (22.22 )
**Supplements taken to reduce pain or improve fitness (%)**			
Calcium	107 (69.93 )	81 (75.00 )	26 ( 57.78)
Vitamin D	115(.75.16 )	77 (71.30 )	38 ( 84.44)
Glucosamine	89 (58.17 )	71(65.74 )	18 (40.00 )
Glucosamine and chondroitin	51 (33.33 )	29 (26.85 )	22 (48.89 )

**Evaluation of biochemical parameters: **A 5-ml blood sample was collected in a plain vial from each patient with MD-OAD before and after the treatment. Blood samples were then centrifuged at 1000×g for 10 min at 4^o ^C to obtain serum. Finally, the serum was used to analyze CK-MM, and aldoA levels for each patient. The biomarkers were rigorously analyzed. CK-MM (U/L) levels were quantitatively assessed using a Creatine Kinase- MM kit (CK-MM/CPK-MM/CK-3) and an immunoassay (Aalto Scientific, Limited, USA). The kit was developed based on the methods reported by Walker (41). AldoA levels (U/L) were quantified using an ALDOLASE (ALS) RX MONZA AD 189 kit (Randox Laboratories Ltd, Antrim, UK) based on a photometric assay at a wavelength of 340 nm. The kit was developed according to the method reported by Thompson and Vignos (42). The subjects suffering from MD-OADs with muscle degeneration and skeletal muscle damage were studied to identify a specific biochemical parameter, such as CK-MM, and aldoA levels, in the affected population

The standard error of the mean (SEM) and their mean differences (MDs), 95% confidence intervals (CIs), and p-values of the two biomarkers such as CK-MM and aldoA and their ratios were evaluated at the baseline and at the end of the six-week treatment. Their percentages of mprovements after the treatment were graphically evaluated.

**Evaluation of pain under Visual analogue scale (VAS): **Visual analogue scale (VAS) for pain is a one-dimensional measure of pain intensity (43). The pain intensity in the last 24 hours was evaluated for each patient at the baseline and at the end of treatment. The percentage of improvements was evaluated at the end of treatment for all the patients separately. Their mean, SD and p-values for overall and separately by gender were also graphically evaluated 

**Evaluation of pain, stiffness and physical function under WOMAC Index: **The scoring data for pain, stiffness and functional disability of individual patient were evaluated using ‘The Western Ontario and McMaster Universities Osteoarthritis Index (WOMAC index) as per method followed by previous researchers (44). The percentage of improvements of pain, stiffness and physical functional abilities were evaluated at the end of treatment for all the patients separately. Their mean, SD and p-values overall and separately by gender were graphically evaluated.

**Evaluation of Karnofsky Performance Status (KPS) score: **Karnofsky performance status (KPS) score is used to determine a patient’s prognosis to carry out daily activities. A higher score indicates the patient is better able to carry out daily activities and its range from 0 to 100 (45). The percentage of improvements was evaluated at the baseline and at the end of the treatment for all the patients separately. Their mean, SD and p-values overall and separately by gender were graphically evaluated.

**Evaluation of Knee-injury Osteoarthritis Outcomes Scale (KOOS): **The Knee-injury Osteoarthritis Outcomes Scale (KOOS) developed by Ewa Roos and co-authors in the 1990s (46) was used to assess the patient’s opinion about their knee and associated problems as an instrument. All the scoring data under KOOS were evaluated for each patient at the baseline and at the end of the six-week treatment. Their mean, SD and p-values overall and separately by gender were also graphically evaluated.

**Evaluation of body mass index (BMI): **Body weight in kilogram was measured without shoes or heavy clothing using an electronic scale. Height in meter was measured without shoes using a wall-mounted stadiometer (47). Body mass index (BMI, kg/m^2^) was calculated for all the patients based on measured weights and heights at pre- and post-treatment. The percentage of improvements was evaluated at the end of treatment for all the patients separately. Their mean, SD and p-values overall and separately by gender were also graphically evaluated.

**Evaluation of Pearson’s correlation coefficients between two biomarkers: **The Pearson’s correlation coefficients between two biochemical markers such as CK-MM at the baseline (CK-MM^b^) and CK-MM after the treatment (CK-MM^t^), aldoA at the baseline (Aldo A^b ^) and aldoA after the treatment (aldoA^t ^), the ratio of (CK-MM^b^ : aldoA^b^) and (CK-MM^t^ : aldoA^t ^) along with their respective p-values were evaluated..

**Evaluation of radiological images** **with the Kellgren and Lawrance system: **Radiological images for both knee joints and lumbo-sacral spine of 153 combined-sex patients, were collected, both anterior-posterior (AP) and lateral views, at pre- and post-treatment evaluations. The AP views of the knee joints of 153 patients are assessed by K-L grading scale (48). The AP view of knee joints and lumbar vertebrae x-ray images of two such patients, (before and after the treatment) are separately evaluated. 

**Evaluation of Indian medicinal plants, their phytoconstituants from aqueous extracts and their established mechanisms of action: **The treatment involves topical application of phytoconstituents from the extracts of seven Indian medicinal plants namely Cissus quadrangularis (whole plant), Calotropis gigantea (root and leaves), Zingiber officinale (rhizome), Rosemarinus officinalis (leaves and flowers), Boswellia serrata (resin), Curcuma longa (rhizome) and Withania somnifera (root) mixed with virgin sesame oil (extracted from seeds at room temperature) and beeswax to make viscous phyto-based oil without using any preservatives or chemicals to preserve the phytochemical properties of plants intact (36-39). 

**Phytotherapeutic treatment protocol: **The treatment protocol is based on well-defined certain principles and theories and also based on the applications of well-known chemical, mechanical, thermal and electrical stimuli which improve the fundamental properties of all muscles such as excitability, conductivity, contractibility, elasticity and viscosity (27-28, 36-37). Each 30 ml of said viscous phyto-based oil prepared from the extracts of seven Indian medicinal plants mixed with virgin sesame oil and beehive-wax is to be applied with the tip of three fingers in particular technique over the skin three times a day with minimum interval of two hours for six weeks ; lying in six different postural positions such as supine, prone, right and left contra-lateral and right and left cross contra-lateral in different programmed sequences to nourish the affected group of badly damaged muscles and nerves in the legs and lumber regions during the disease. Therefore, the present treatment protocol has been followed the earlier studies (27, 28, 36-37, 49-55).

**External study reviewers: **All results and data before and after the treatment were evaluated by an external reviewing panel, not in contract with the registry patients. 

**Data collection and Statistical analysis: **Data were summarized using descriptive statistics for continuous variables (e.g., mean, standard deviation, standard error of the mean, number of patients, minimum, maximum), frequency tables, or ratios for discrete variables, and 95% confidence intervals. Statistical analyses were done using software (Graph Pad Prism, Version5.0) with repeated measures for student-t test to determine the significant values at p<0.05 level along with r (Pearson’s correlation coefficient) values to determine strong and weak correlation among two variables for measuring different improvement parameters of combined-sex, female and male patients separately. An alpha level of 5% was established i.e., a p-value less than 0.05 was considered statistically significant.

## Results

**Enrolment and baseline characteristics of patients: **A total of 153 patients aged 59.89±11.37 years (71% women) were included in the study. All the patients were suffering from MD-OAD for 7.89±1.90 years having massive muscle wasting specially in the thigh and calf regions, bone erosion and skeletal muscle damaged confirmed by the serum tests of CK-MM and aldoA and radiological images. The baseline demographic characteristics of all patients are shown in table 2. 

**Biochemical parameters:** Table 3 shows that the mean levels of CK-MM and Aldo were reduced to their normal limits ( CK-MM: for male <171 and female <145 U/L and aldoA: <7.6U/L and their differences and the ratios of CK-MM and aldoA at the baseline (CK-MM^b^: aldoA^b^) and at the end of six-week treatment (CK-MM^t^: aldoA^t^) were highly significant (p<0.0001) when compared to the baseline for both overall and separately by gender.

**Table 3:** Statistical analysis of CK-MM, aldoA and ratio of CK-MM and aldoA of 153 patients

**Biochemical parameter**	**Gender**	**Baseline**	**End of 6-week**	**Improvements on elevated levels of biomarkers at the end of 6-week**			
				**MD**	**95% CI of difference**		**P-value**
		**Mean (SEM)**	**Mean (SEM)**		**Lower limit**	**Upper limit**	
CK-MM (U/L)	Female (n=108)	206.45 (8.207)	77.99 (1.230)	128.46	112.10	144.82	<0.0001
	Male (n=45)	252.97 (15.408)	94.24 (2.697)	158.73	127.64	189.81	<0.0001
Aldo A U/L)	Female (n=108)	7.25 (0.264)	4.91 (1.230)	2.34	1.77	2.91	<0.0001
	Male (n=45)	6.70 (0.276)	5.02 (0.201)	1.68	1.00	2.36	<0.0001
Ratio of CK-MM & AldoA	Female (n=108)	30.50 (1.172)	17.05 (0.542)	13.45	10.90	15.99	<0.0001
	Male (n=45)	40.42 (2.785)	20.76 (1.425)	19.66	13.44	25.88	<0.0001

Table 4 shows the levels of correlation coefficients between CK-MM at the baseline (CK-MM^b^) and at the end of treatment (CK-MM^t^), were not significant (P=0.024, P=0.574 and P=0.434, respectively) for combined-sex patients, female-only and male-only patients. 

The correlation coefficients between aldoA at the baseline (aldoA^b^) and at the end of treatment (Aldo A^t^) and the ratio of CK-MM and Aldo A at the baseline (CK-MM^b^ : AldoA^b^) and at the end of 6-week of treatment (CK-MM^t^ : aldoA^t ^) were all highly significant (P=0.000) both overall and separately by gender. 

The correlation coefficients between CK-MM at baseline (CK-MM^b^) and aldoA at the baseline (aldoA^b^) were all highly significant (p<0.05). 

The correlation coefficients between CK-MM at baseline (CK-MM^b^) and aldoA at end of treatment (aldoA^t^) were not significant (P=0.353, P=0.228 and P=0.828, respectively) for combined-sex patients, female-only patients and male-only patients. 

**Figure 1:**
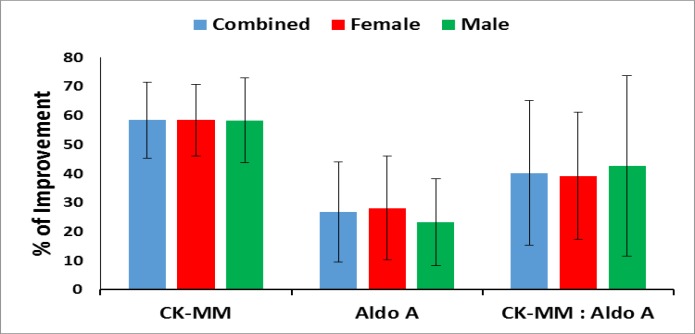
Percentage of improvement of biomarkers CK-MM and aldoA and their ratios at the end of the 6-week treatment (*p<0.0001)

The percentage of improvements of CK-MM and aldoA after the end of treatment were highly significant (p<0.0001) both overall and separately by gender and shown in figure 1.

**Figure 2:**
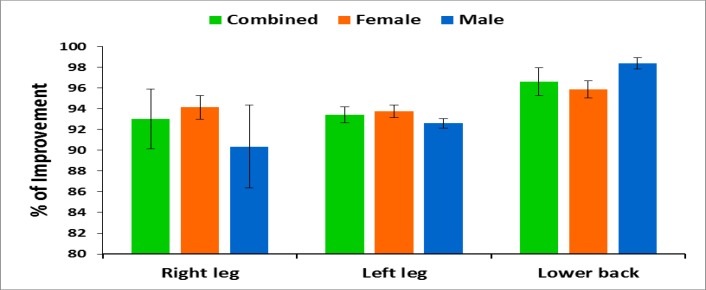
percentage of Improvement of pain under VAS after 6-week treatment (*p<0.0001)

**Figure 3:**
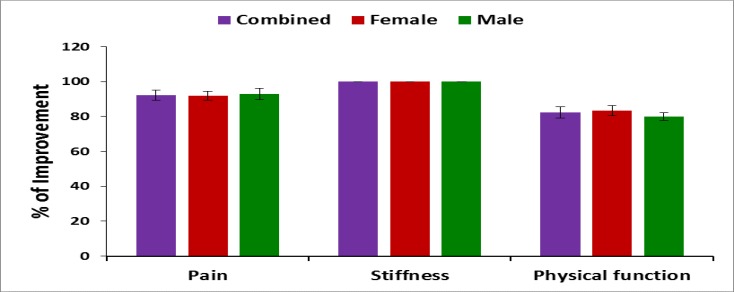
% of Improvement of pain, stiffness & physical function under WOMAC index after the 6-week treatment (*p<0.0001)

**Figure 4:**
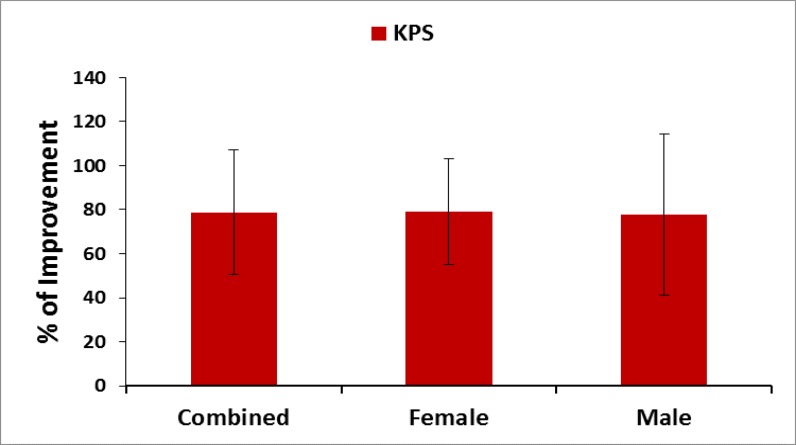
% of Improvement of pain under KPS after 6-week treatment (*p<0.0001)

**Figure 5:**
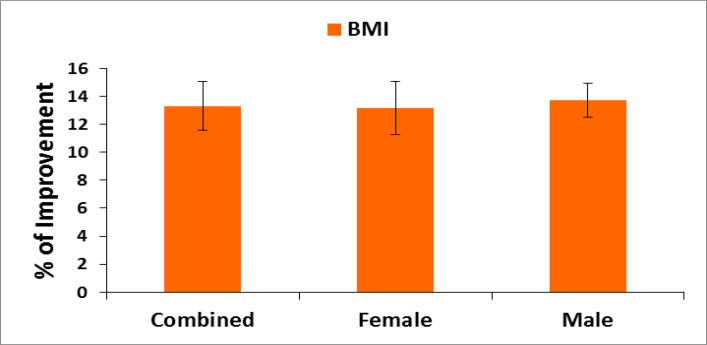
% of Improvement on reduction of body weight as assessed by BMI after 6-week treatment (*p<0.0001)

**Figure 6:**
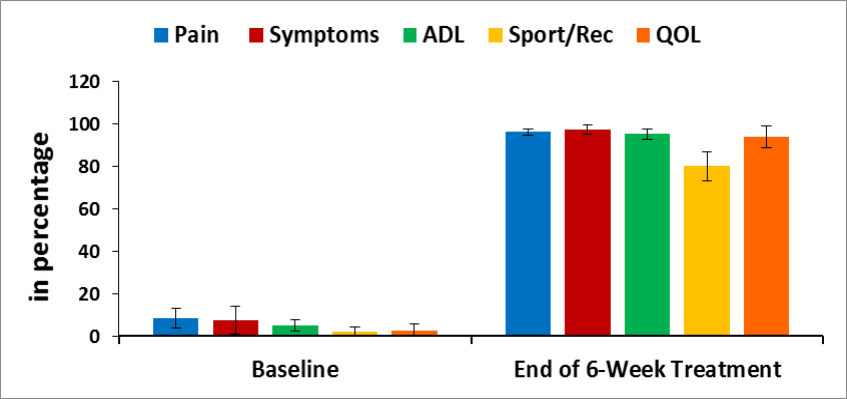
KOOS Profiles before and after the treatment of six weeks by phytotherapy. Mean KOOS score (n=153) at the baseline and at the end of six weeks assessment (*p<0.0001)

**Pain, stiffness, performance parameters and BMI: **The percentage of improvements: on pain under VAS; on pain, stiffness and physical functions under WOMAC index; on performance on daily activities under KPS; of the five separately scored subscales under KOOS knee survey and reduction of body weight confirmed by BMI after the treatment for combined-sex, female-only and male-only patients were all highly significant (p<0.05) both overall and separately by gender depicted in figures 2-6.

**Table 4:** Analysis of correlation coefficients of biomarkers between baseline and end of 6-week treatment

**Gender**	**CK-MM** ^b^ ** vs. ** **CK-MM** ^t^		**Aldo A** ^b ^ ** vs. ** **Aldo A** ^t^		**CK-MM** ^b^ ** vs. ** **Aldo A** ^b^		**CK-MM** ^t ^ ** vs. ** **Aldo A** ^t^		**CK-MM** ^b^ **: Aldo A** ^b ^ ** vs. CK-MM** ^t^ **: Aldo A** ^t^	
	**R-value**	**P-value**	**R-value**	**P-value**	**R-value**	**P-value**	**R-value**	**P-value**	**R-value**	**P-value**
Female-only (n=108)	0.055	0.574	0.638	0.000	0.286	0.002	0.117	0.228	0.438	0.000
Male-only (n=45)	-0.120	0.434	0.679	0.000	0.105	0.494	-0.033	0.828	0.444	0.000

CK-MM^b^: baseline data of CK-MM,

CK-MM^t^: data after treatment of CK-MM,

Aldo A^b^: baseline data of aldoA,

aldoA^t^: data after treatment of aldoA

**Figure 7:**
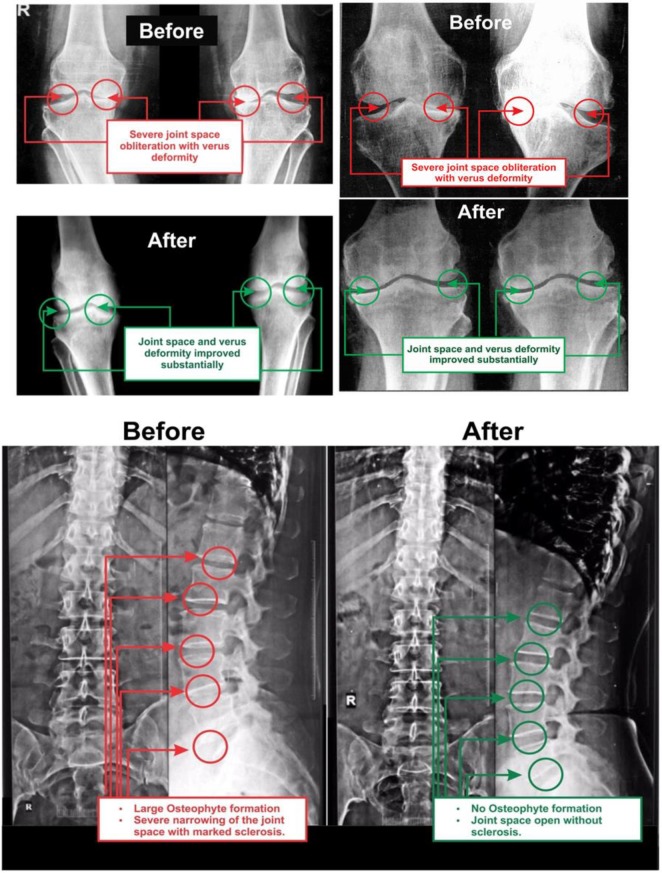
AP views of x-ray for knee-joint and lumbar vertebrae before and after the treatment

**Table 5:** Statistical analysis of anatomical features of 153 patients at post treatment

**Anatomical parameter**	**Gender**	**Baseline**		**End of 6-week**		**Improvement on elevated level of anatomical parameters at the end of 6-week**			
		**Mean (SEM)**		**Mean (SEM)**		**Right Leg**		**Left Leg**	
		**Rt.**	**Lt.**	**Rt.**	**Lt.**	**MD (95%CI)**	**P-value**	**MD (95%CI)**	**P-value**
**SLR (degree)**	**Female (n=108)**	54.03 (1.015)	54.9 (0.945)	75.91 (0.359)	75.91 (0.359)	-21.88 (-24.00, -19.76)	<0.0001	-21.01 (-23.00, -19.02)	<0.0001
	**Male (n=45)**	50.78 (1.513)	51.82 (1.511)	74.82 (0.626)	74.82 (0.626	-24.04 (-27.29, -20.78)	<0.0001	-23.00 (-26.25, -19.75)	<0.0001
**KFS (degree)**	**Female (n=108)**	112.72 (0.723)	113.21 (0.652)	143.20 (0.185)	143.20 (0.185)	-30.48 (-31.95, 29.00)	<0.0001	-29.99 (-31.33, -28.65)	<0.0001
	**Male (n=45)**	112.60 (0.961)	112.02 (1.030)	142.76 (0.283)	142.76 (0.283)	-30.16 (-32.15, -28.17)	<0.0001	-30.74 (-32.86, -28.62)	<0.0001
**KES** **(degree)**	**Female (n=108)**	17.50 (0.215)	17.39 (0.182)	8.76 (0.050)	8.76 (0.050)	8.70 (8.30, 9.17)	<0.0001	8.63 (8.26, 9.00)	<0.0001
	**Male (n=45)**	17.56 (0.280)	17.76 (0.186)	9.66 (0.133)	9.66 (0.133)	7.90 (7.28, 8.52)	<0.0001	8.10 (7.64, 8.55)	<0.0001

**Anatomical parameters:** Table 5 shows that the mean ±SEM values of SLR and KFS at post-treatment all increased and that decreased for KES and observed to be all symmetrical for both the legs. All the differences were highly significant (p<0.0001) when compared to pre-treatment for both overall and separately by gender.

The overall measurements of KGB, DAP and DBP all reduced and DCM and DTM were all increased and observed to be symmetrical for both the legs at the end of the 6-week treatment and were also highly significant (p<0.0001) when compared to the baseline [data not shown]. 

**Improvement on muscular strength during MD-OAD as per radiological image assessed by K-L grading scale: **All the anterior-posterior (AP) views of the x-ray reports of 153 patients with OAD at the baseline exhibited degenerative changes, particularly in the medial tibio-femoral compartment, with marked joint space narrowing and bilateral varus/valgus deformities. Some cases exhibited near-complete medial compartment joint space obliteration and also degenerative changes with osteophytes in the lumbar vertebrae. The AP view of x-ray for bilateral knee joints and lumbar vertebrae of patients after six-week treatment indicated no such degenerative changes and assessment under K-L grading scale shown in **table-6**. x-ray images of two such patients suffering from pain in the knee joint and lumbar region before and after the treatment are depicted in figure 7.

## Discussion

The present results indicated that MD-OADs can easily be recognized by elevated biomarkers such as CK-MM and aldoA and their ratio. Major research works have been reported that elevation of two biochemical markers such as CK-MM and aldoA are important to detect the muscular dystrophy and skeletal muscle damage (17-20, 22, 27-28, 36-38). There is currently no way to prevent or reverse MD-OAD but frequently different medications are used for temporary relief such as NSAIDs to prevent pain and inflammation but have tremendous side-effects like cardiovascular and gastrointestinal problems and corticosteroids, which may help to increase muscle strength and slow progression but can weaken bones and increase weight gain for long-term use and use of other kinds of therapies as mentioned earlier, which may improve the delay of progression of symptoms, thus, the quality of life may be saved temporarily. In the present study, males were observed more susceptibly than females on biomarker evaluation but earlier study revealed that women are more vulnerable than men (9, 11, 13). 

Simultaneously, the declining level of these two biomarkers along with improved deranged anatomical features, pain parameter under VAS, improvement of physical functional abilities and quality of life under WOMAC index, KPS scale, KOOS and lowering obesity under BMI by topical phytotherapeutic treatment protocol for a period of 6 weeks, was an endeavour in the present study. Generally, phytomedicines from medicinal plants derived compounds are well-established for the prevention of arthritis, obesity, oxidative stress, inflammation, neuro-degenerative diseases, etc. (36-38, 49, 53-55) It was observed in the present study that both the biomarkers decreased after topical application of aqueous extract of medicinal plants as per research protocol developed by Ganguly et al. (36-37) and Ganguly (38), followed by Belcaro et al. (39). Both the levels of the biomarkers and their ratios were substantially reduced at the end of the six-week treatment in the case of percentage improvement study done separately for combined-sex, female-only and male-only patients (Table 3 and Figure 1). The author had already proven the efficacy of the topical phytotherapeutic treatment protocol by exhibiting 42 pairs of knee-joints of 21 patients before and after the treatment irrespective of age and sex and even after failed total knee surgery where all the patients were suffering from MD confirmed with their serum test of CK-MM and aldo A (36-38, 49, 53-55) .

The percentage improvement for the study of pain and related parameters were substantially achieved under the well-known guidelines for the standard clinical outcomes such as VAS, WOMAC Index, KPS, and KOOS and there is a reduction of body weight as assessed by BMI at the end of the six-week phytotherapy treatment for all the studied subjects. The result from the deranged anatomical parameters shows that there are substantially increasing and decreasing phenomenon of the group of muscles connected with various joints and both legs were symmetrical in respect of the measurements of KGB, DAP, DBP, DCM, DTM, SLR, KFS and KES at six weeks post-treatment which indicate the improvement of muscle wasting, muscle weakness and degeneration that occurred during MD-OAD at the baseline. Several researchers have emphasized the normalization of the above-mentioned parameters along with the prevention of osteoarthritis in human (36-39) but the present study indicates aqueous extracts of medicinal plants and their phytoconstituents may participate individually and/or combination repair MD in relation to the normalization of biochemical markers. The correlation coefficient interactions highly improved after the treatment when compared to baseline data. It is well-known that the increased levels of CK-MM and aldo A in the blood of human indicate muscle wasting and skeletal muscle damage occurred in the tissue (22, 51-52, 54) in which abnormal muscle anatomy, pain and degenerative changes in bones were found (54). The present study revealed that the normalization of MD-OAD of patients is possible with the help of specialized phytotherapeutic treatment protocol by the evidence of declining levels of CK-MM (for female <145 U/L and for male <171) and aldoA (<7.6 U/L) followed by the normalization of anatomical parameters along with the repair of OADs identified in biomarkers, recovery of pain, muscle stiffness, and normal physical day to day functional activities and reduction of body weight as assessed by the standard international clinical outcomes such as VAS, WOMAC index, KPS, KOOS and BMI together with the confirmed improvement in radiological images of joints. 

Moreover, the few research works have been emphasized on structural and functional abnormalities of dystrophin protein located within sarcolemma and causative factor of MD (12) but the exact mechanisms of MD-OADs are not clearly understood and researcher investigated only muscle damage during knee osteoarthritis (13). In conclusion, It is firmly concluded from the results that for the first time, normalization of MD-OAD patients with a unique method of topical phytotherapeutic treatment technique for 6 weeks is confirmed by assessing the decreased levels of CK-MM and aldoA along with improved deranged anatomical features, diminished levels of pain, stiffness, increased the quality of life and decreased body weight as assessed by VAS, WOMAC Index, KPS, KOOS and BMI together with improved radiological images correlated with K-L grading scale. Future research should be carried out to detect the structural and functional abnormalities of dystrophin protein in serum during MD-OADs.

Limitation of the study: The patients suffering from chronic disorders are restricted to this present study such as Duchenne muscular dystrophy (DMD); Becker muscular dystrophy (BMD); rheumatic diseases; osteochondritis diseases; congenital dysplasia; radicular syndrome; joint symptoms caused by malignant tumors; dermatomyositis and polymyositis diseases; iliopectineal or trochanteric bursitis; ischemic bone necrosis; bone and joint infectious diseases; cuts, wounds or any type of chronic skin and infectious diseases; parallel multiple drug dependence for concomitant diseases or risk conditions requiring drug treatment including psychiatric diseases; a history of cancer, including caranomatosis and granulocytic leukemia; a history of severe neurological diseases; a history of chronic liver, kidney and heart diseases and also patients who did not agree to give blood sample, may be due to drugs/alcohol addiction, pregnancy and such other reasons.
